# Evaluation of Prevalence, Homology and Immunogenicity of Dispersin among Enteroaggregative *Escherichia coli* Isolates from Iran

**DOI:** 10.6091/.21.1.40

**Published:** 2017-01

**Authors:** Mohammad Reza Asadi Karam, Ali Akbar Rezaei, Seyed Davar Siadat, Mehri Habibi, Saeid Bouzari

**Affiliations:** 1 Department of Molecular Biology, Pasteur Institute of Iran, Tehran, Iran; 2 Department of Mycobacteriology and Pulmonary Research, Pasteur Institute of Iran, Tehran, Iran

**Keywords:** Enteroaggregative *E. coli*, Diarrhea, Enzyme-linked immunosorbent assay

## Abstract

**Background:**

Diarrhea, caused by enteroaggregative *Escherichia coli* (EAEC), is an important infection leading toillness and death. Numerous virulent factors have been described in EAEC. However, their prevalence was highly variable among EAECs of distinct geographic locations. Studies have shown that dispersin (antiaggregation protein, *aap*) is one of the important and abundant virulent factors in EAEC. In this study, we aimed to determine the presence, conservation, and immunogenicity of *aap* gene in EAEC isolated from Iranian patients.

**Methods:**

PCR amplification of *aap* gene in the EAEC isolates was performed, and the *aap* gene was cloned in pBAD-gIIIA vector. The sequence of *aap* gene was analyzed using the ExPASy and BLAST tools. The expression of *aap* gene was performed in *E. coli* Top10, and expression confirmation was carried out by SDS-PAGE and Western-blot techniques. Rabbits were immunized with purified dispersin protein emulsified with Freund’s adjuvant. Sera were collected and examined for antibody response. Finally, *in* *vitro* efficacy of dispersin and anti-dispersin was evaluated.

**Results:**

The results of PCR showed the presence of *aap* gene in all of the EAEC isolates with significant homology. Finally, the significant difference between the levels of IgG response in dispersin-injected rabbits and control group was observed.

**Conclusion:**

Our results were in accordance with other studies that reported the presence of dispersin in the EAEC isolates with high conservation and immunogenicity. Hence, dispersin could be a promising candidate for any probable prevention against EAEC infections.

## INTRODUCTION

Diarrhea is a predominant cause of mortality and morbidity in developing world, especially among children under five years of age[1,^2^]. Diarrheagenic *Escherichia coli* strains are major etiological causes of diarrhea. Based on the clinical and epidemiological features and specific virulent factors associated with specific serotypes, these strains are divided into six major pathogenic types, including enteroaggregative *E. coli* (EAEC), enteropathogenic *E. coli*, enteroinvasive *E. coli*, enteroheamorrhagic *E. coli*, enterotoxigenic *E. coli* and diffusely adherent *E. coli*[^2^,^3^]. These strains have also acquired specific virulence factors that increase their ability to cause a broad spectrum of diseases by different mechanisms[4].

EAEC is an emerging diarrheal pathogen. The increasing numbers of EAEC causing acute, persistent and traveler’s diarrhea have been associated with contaminated food and water[1,^2^]. EAEC has the ability of adhering to HEp2 cells in a stacked brick formation, named aggregative adherence (AA). The AA phenotype is encoded by 55–65 MDa plasmids, called pAA, on which a number of virulence genes are placed[^2^,^5^]. These genes include the AA fimbrial (AAF) genes (AAFI, AAFII and AAFIII), the transcriptional activator gene (*aggR*), antiaggregation protein gene (*aap*), plasmid-encoded toxin (*pet*) and antiaggregation protein transporter gene (*aat*) [1,^2^,^4^].

Dispersin is a low-molecular-weight protein encoded by *aap* and regulated by *aggR* gene in EAEC strains[6]. Dispersin is secreted to the surface of EAEC strains and binds to the lipopolysaccharide (LPS) in the outer membrane[1]. Recent studies have suggested that dispersin neutralizes the charge of the LPS and AAFs, which results in the release and binding of dispersin to the surface of intestinal mucosa[1,7]. Therefore, dispersin decreases bacterial autoaggregation allowing dispersion on the intestinal mucosa[1].

Due to the occurrence of diarrheal infections caused by EAEC strains, development of an efficacious vaccine would have a significant impact on controlling the spread of the disease and reduces the healthcare costs. Some antigens of the bacterium have been tested as vaccine candidates with limited success[^2^, 8]. Thus, to achieve an ideal target for prevention of EAEC infections, evaluation of the other vaccine antigens of EAEC strains is needed.

Investigations have indicated that dispersin is one of the most abundant and immunogenic components of the EAEC strains, suggesting that this protein can be considered as a suitable candidate for the prevention of the EAEC infections[6,9]. In the current study, we isolated and expressed the dispersin of EAEC isolates of Iranian patients and then assessed the immunogenicity of dispersin in an animal model.

## MATERIALS AND METHODS

### Patients and clinical specimens

Clinical samples used in this study were collected from patients at several hospitals in Tehran, Iran. All specimens were obtained according to the standard procedures and stored at -80°C. All stool samples were cultured on blood and MacConkey agar (Merck, Germany) at 37°C for 24 h. Bacterial identification was performed by the routine culture method and biochemical tests[10].

### DNA extraction and PCR amplification

All EAEC samples were grown in 5 ml Luria-Bertani (LB) broth medium[10]. The genomic DNA was extracted using the phenol-chloroform method[10]. PCR amplification of *aap* gene was carried out using specific primers (*aap*-forward: CATGCCATGGCAA TGAAAAAAATTAAG and *aap*-reverse: GCTCTAG AGCTTTAACCCATTCGG), which were designed based on the *aap* gene sequence of EAEC 042 strain (GenBank accession no. NC_017627.1). PCR reactions were set in 50-µl volume containing 2 µl DNA template, 5 µl 10× reaction buffer, 2 µl dNTPs (10 mM), 2 µl MgCl_2_ (50 mM), 2 µl of each primer (10 pmol), and 1 U *pfu* DNA polymerase (Fermentas, Germany). The PCR conditions included an initial denaturation at 94°C for 3 min, followed by 30 cycles at 94°C for 60 s, 55°C for 60 s and 72°C for 60 s, with a final extension step at 72°C for 8 min. After the amplification of *aap* gene, 5 µl of the samples was subjected to electrophoresis on 1% agarose gel (Sigma, USA) to confirm the presence of the amplified products.

### Cloning and analysis of nucleotide sequence of aap gene

PCR amplification of *aap* gene was performed using primers designed to introduce *Nco*I site at the 5´-terminus and *Xba*I site at 3´-terminus of the gene. In the next stage, the PCR products were gel-purified using the gel extraction kit (Roche, Germany) and digested with the restriction enzymes (Fermentas, Germany). The digested products were cloned into the *Nco*I and *Xba*I sites of plasmid expression vector pBAD/gIIIA containing histidine tag sequence to generate proteins fused with the tag at the C-terminal. The ligated plasmid was then transformed into the competent *E. coli* Top10 (Invitrogen, USA) according to the manufacturer’s instructions. The selected clones were analyzed by gel electrophoresis, PCR, restriction analysis and sequencing (Eurofins MWG service, Germany). Nucleotide and deduced amino acid sequences of *aap* gene were analyzed using the ExPASy Proteomics (http://www.expasy.org/tools/) and BLAST (blast.ncbi.nlm.nih.gov) tools.

### Expression and evaluation of recombinant proteins

Recombinant *E. coli* Top10 cells were grown in LB medium containing ampicillin (100 µg/ml) at 37°C overnight. In the next day, 500 ml LB medium was inoculated with 5 ml Top10 cells cultured overnight. The inoculated cultures were grown with agitation under aerobic conditions at 37°C to reach OD600nm~0.5. Then the expression of the cloned genes was induced by different concentrations of arabinose (final concentration 0.002-20%). After incubation for 4 h, the cells were harvested by centrifugation at 4°C for 15 min and stored at -20°C until further use.

To analyze the expression of proteins by sodium SDS-PAGE, the bacterial pellets were suspended in a loading buffer and heated at 95°C for 5 min. Then 30 µl of each sample was subjected to 12-15% SDS-PAGE gels. For Western-blot analysis, the samples were separated by SDS-PAGE and transferred to nitrocellulose membrane (Schleicher and Schuell, Germany) using a liquid transfer system (Bio-Rad, USA). Membranes were blocked with skim milk (Merck, Germany) in PBST (PBS 1%+Tween20) and then washed with PBST. The membranes were incubated with the horseradish peroxidase-conjugated His-tag antibody (Invitrogen, USA), and the positive signals were detected by DAB (3,3′-diaminobenzidine) (Invitrogen, USA) and H_2_O_2_ as substrate.

### Purification of the recombinant proteins

For protein purification, cell pellet was harvested and lysed in a denaturation buffer. The supernatant was collected and applied to a Ni-NTA column (Qiagen, Germany). Also, a single-step protocol was used to purify and remove the endotoxin of the proteins on the column[11]. After dialysis, the identity and the purity of the eluted proteins were estimated by SDS-PAGE and Western-blotting. Endotoxin (LPS) level of the proteins was determined by using the Chromogenic LAL test (Lonza, USA) as described by the manufacturer. The level of the purified proteins was quantified by the Bradford’s assay (Bio-Rad, USA).

### Immunization and generation of antibody against dispersin

Female New Zealand rabbits, obtained from Pasteur Institute of Iran, Karaj, were housed in standard Plexiglas cages with free access to food (standard laboratory rodent′s chow) and water. All animal experiments were carried out in accordance with the European Communities Council Directive of 24 November 1986 (86/609/EEC). For dispersin antibody production, three New Zealand rabbits were first immunized subcutaneously with 400 μg purified dispersin protein emulsified with Freund’s complete adjuvant (Sigma, USA) and boosted twice with 400 μg protein in Freund’s incomplete adjuvant (Sigma, USA) at 2-week intervals. Sera were collected two weeks after each immunization by centrifugation of blood cells at 5000 ×g for 15 min and examined for antibody responses.

### Analysis of antibody responses by ELISA

Antibody response to recombinant dispersin protein was evaluated by ELISA as described previously[12]. Briefly, 96-well plates were coated with purified dispersin diluted in PBS and incubated at 4°C overnight. Wells were then blocked with 3% bovine serum albumin (Sigma, USA) and incubated with serial dilutions of immune sera starting from 1:50. The plates were then incubated with horseradish peroxidase-conjugated goat anti-rabbit IgG (Sigma, USA), and the signals were developed by 3,3,5,5-tetramethyl-benzidine substrate. The reaction was stopped, and the absorbance was read on an ELISA plate reader (BioTek technology, Germany) at 450 nm.

### In vitro assay of dispersin and anti-dispersin antibody

The ability of anti-dispersin antibody to inhibit the adherence of EAEC strains to HeLa cell lines (National Cell Bank, Pasteur Institute of Iran, Tehran) was determined. HeLa cells (10^5^ per well) were grown in 6-well microtiter plates (Nunc, Germany) containing microscopic coverslips in a RPMI medium (pH 7.4, supplemented with 10% heat inactivated fetal bovine serum and 100 units/ml penicillin-streptomycin) in a 5% CO_2_ atmosphere at 37ºC for 24 h. Next, the supernatant cells were removed, and DMEM medium containing Di-mannose was added. Then 50 µl serum from the immunized rabbits was mixed with 50 µl overnight culture of EAEC isolates, and the mixture was transferred to the plates and incubated at 37ºC for 3 h. Thereafter, the cells on the coverslips were washed with 1% PBS and fixed with 1% acetic acid in 50% ethanol for 15 min. The fixed cells were stained with Giemsa stain and visualized under a microscope to evaluate the adherence or dispersion pattern of the EAEC isolates. EAEC strain 042 and *E. coli* k12 were used as positive and negative controls, respectively.

### Statistical evaluations

The one-way ANOVA, student’s *t* test and Tukey’s HSD test were used to compare the differences between the mean values of the groups using SPSS software (version 16.0). In all experiments, *P*<0.05 was considered statistically as significant.

## RESULTS

### Gene amplification and sequence analysis

**Fig. 1:**
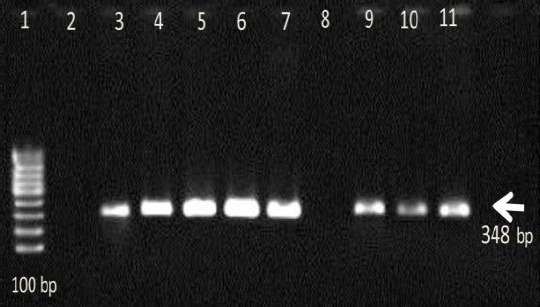
PCR amplification of *aag* gene. The genome of the enteroaggregative *Escherichia coli* (EAEC) isolates was extracted, and PCR amplification of *aap* gene was performed by PCR method. Lane 1, molecular weight marker (100 bp ladder DNA); Lane 2, *E. coli* K12 strain (negative control); Lane 3, EAEC strain 042 (positive control); Lanes 4-7 and 9-11, products of *aap* gene (about 348 bp); Lane 8, negative control (double-distilled water).

Twenty EAEC were isolated from the stool culture of patients in Tehran, Iran and confirmed by routine laboratory methods. The genomic DNA of the EAEC isolates was extracted and used to perform PCR assay. The results of PCR showed that *aap* gene was present in all of the analyzed EAEC isolates, and the length of PCR fragment of *aap* gene was approximately 348 bp (Fig. 1).

**Fig. 2:**
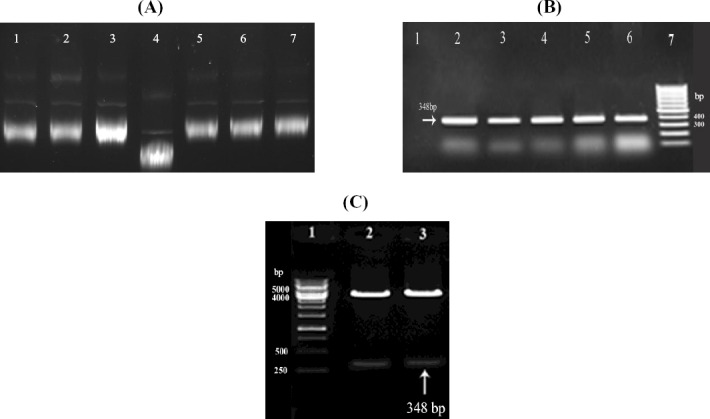
Confirmation of cloning of *aap* gene in expression vector pBAD-gIIIA. The *aap* genes were cloned in pBAD-gIIIA vector and the confirmation of cloning was carried out by (A) electrophoresis (Lanes 1-3 and 5-7, pBAD-aap clones; Lane 4, pBAD-gIIIA); (B) PCR Lane 1, negative control; Lanes 2-6, pBAD-aap clones; Lane 7, 100 bp molecular weight marker; (C) Enzyme digestion with *Nco*I and *Xba*I restriction enzymes (Lane 1, molecular weight marker (1-kb ladder mix); Lanes 2 and 3, pBAD-aap clones).

For sequencing, the *aap* gene was cloned in expression vector pBAD-gIIIA. To Confirm the cloned fragment, PCR and digestion with *Nco*I and *Xba*I restriction enzymes were carried out, and the results are shown in Figures 2A, 2B and 2C, respectively. The comparison between *aap* sequences and published sequences of the gene using BLAST and ExPASy tools indicated significant homology of ≥98%, confirming that *aap* is highly conserved among the isolates. Furthermore, *aap* sequences of our isolates showed 100% homology in all isolates. The determined sequence of this *aap* gene was submitted to GenBank and has been assigned under accession number of KJ028043.

### Expression and purification of recombinant proteins

**Fig. 3:**
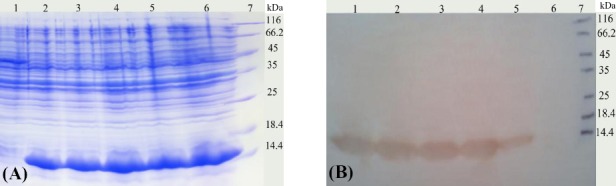
Analysis of the expressed products of *aap* gene by SDS-PAGE and Western-blotting. The expressed protein dispesin (*aap*) was evaluated by (A) SDS-PAGE (Lane 1, uninduced construct; Lanes 2-6, *aap* induced by arabinose 0.002%, 0.02%, 0.2%, 2% and 20%, respectively; Lane 7, protein marker); (B) Western-blot with His-tag monoclonal antibody (Lanes 1-5, *aap* induced by arabinose 0.002%, 0.02%, 0.2%, 2% and 20%, respectively; Lane 6, uninduced construct; Lane 7, protein marker).

Optimum expression was obtained with different concentrations of arabinose (0.002-20%) with incubation time of 4 h. The results of SDS-PAGE and Western-blot analysis from bacterial clones expressing the dispersin protein are shown in Figures 3A and 3B, respectively. SDS-PAGE and Western-blotting using anti-His-tag antibody confirmed the presence of a single band of ~12 kDa.

**Fig. 4:**
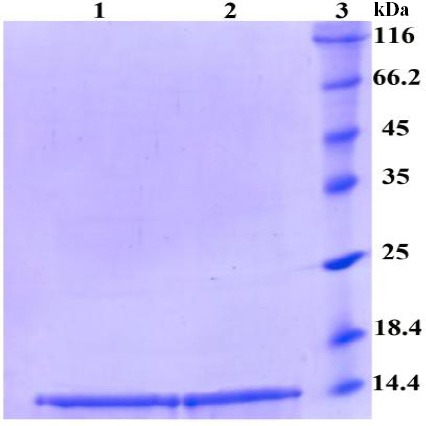
Analysis of purified dispersin protein by SDS-PAGE. Lanes 1 and 2, purified dispersin protein (elution 1 and 2, respectively); Lane 3, protein marker.

The His-tagged dispersin was purified by affinity chromatography using Ni-NTA column under native conditions. The purity of the recombinant protein was checked by SDS-PAGE (Fig. 4) and Western-blot analysis (data not shown). Endotoxin levels of the purified proteins were ≤0.02 EU/ml, as determined by Limulus amebocyte lysate test. The level of the purified proteins was about 1 mg/ml as measured by the Bradford’s assay.

### Evaluation of antibody response

**Fig. 5:**
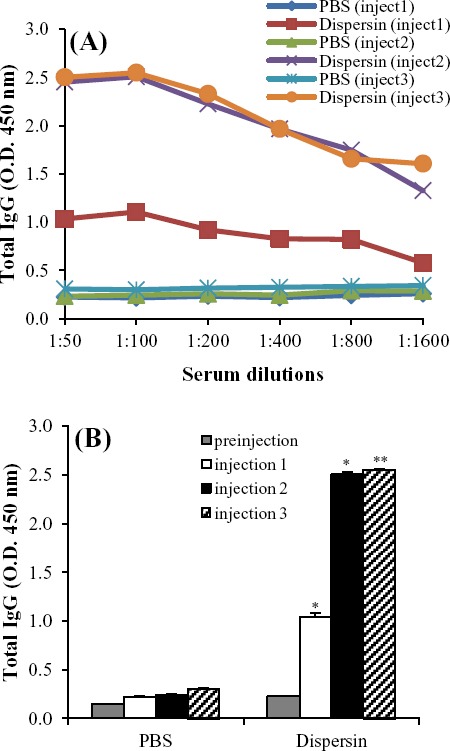
Induction of anti-dispersin IgG responses in rabbits. Rabbits were immunized three times with dispersin and admixed with Freund’s adjuvat. The control groups were immunized with PBS. (A) The IgG response after the first, the second and the third immunization. (B) The dispersin-specific IgG profile after the third immunization at 1:100 serum dilution. * indicates statistical significance between the first injection and the control (*P*<0.05). ** indicates statistical significance of the second and the third injection in comparison to the control group (*P*<0.01). Preinjection is the result of anti-dispersin IgG response in serum collected before the first injection of dispersin and was used as a control serum. Results were expressed as mean and standard errors.

To evaluate the ability of dispersin to promote a humoral immune response, rabbits were immunized with dispersin antigen in Freund’s adjuvant. Our results showed that IgG response was detected after the first injection in all rabbits compared to the control rabbits, with an increase trend after the second and the third injection (Fig. 5A). At first immunization, a significant difference was observed between the IgG levels in immunized rabbits and control group that continued after the second and the third injection, while the IgG level was not different significantly between the second and third immunization (Fig. 5B) (*P*>0.05).

### In vitro evaluation of dispersin and anti-dispersin

**Fig. 6:**
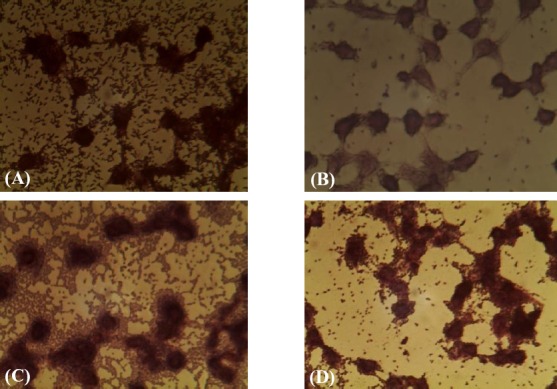
Evaluation of dispersin and anti-dispersin antibody in HeLa cell. (A) EAEC 042 strain as a positive control; (B) E. coli K12 strain as a negative control; (C) An EAEC isolate in the absence of anti-dispersin antibody; (D) An EAEC isolate in the presence of anti-dispersin antibody.

We evaluated the effect of anti-dispersin antibody on inhibition or dispersion of the EAEC isolates in HeLa cells. Our results indicated that in the absence of anti dispersin antibody, about 80% of the analyzed EAEC isolates had high tendency to adhere to the HeLa cells; however, in the presence of anti-dispersin antibody, the majority of EAEC isolates demonstrated a decrease in adherence to the cells (Fig. 6).

## DISCUSSION

Despite development in detection and treatment of diarrheagenic pathogens, it has been reported that diarrhea causes about 1.6-2.5 million deaths annually[^2^,^13^]. Diarrheagenic *Escherichia coli* are responsible for the majority of diarrhea cases, which among them EAECs are important causes of diarrhea in both industrialized and developing countries[1, 4]. For example, one large prospective surveillance study in the USA has indicated that EAEC strains are the most common causative agents of diarrhea in all age groups[1]. Other studies have shown that EAEC strains are among the important diarrheagenic strains isolated from Iranian patients[14-18]. On the other hand, treatment of the EAEC infections has become increasingly difficult due to the emergence of resistant pathogens[^2^]. Hence, the management of EAEC infections needs to further attention. In this regard, development of an effective vaccine to protect against these infections would be enormously beneficial for human health[^2^].

To date, there has been limited success in developing an efficacious vaccine against EAEC infections. Therefore, the limited success of previous vaccines highlights the need for evaluation of new target antigens of EAEC strains to develop an efficacious vaccine against EAEC. Ideally, the target antigen should be highly immunogenic, highly conserved, prevalent among clinical isolates and surface-exposed[19].

Dispersin is one of the major virulence factors of EAEC pathogens that is responsible for dispersing and penetration of EAEC across the intestinal epithelium. Dispersin contains N-terminal epitopes that are involved in diffusion of infection. Also, this protein carries some conserved regions, which can serve as diagnostic antigens[1,6,7]. The analysis of *aap* gene encoding dispersin has shown that neither the nucleotide sequence of *aap* nor its deduced amino acid sequence displays significant identity to any other protein or gene in the databases.

Using PCR and sequencing, we assessed the conservation of the *aap* gene among EAEC strains isolated from Iranian patients. Our results indicated the presence and highly conservation of dispersin (*aap*) amino acid sequences in all of the isolates. In a study, Monteiro *et al*.[20] compared the alignment of the 94-amino-acid sequences of dispersin with the sequence of the prototype strain 042, and the result demonstrated a high amino acid identity level of 93%-95%. Also, the *aap* sequences showed high homology to each other, suggesting the existence of a shared *aap* allele in these strains. In other study by sheikh *et al*.[6], 80% of EAEC isolates were identified as carriers of the *aap* gene. Jenkins *et al*.[21] have also indicated that *aap* gene was present in high proportion (98%) in EAEC strains isolated from diarrheal patients.

Our animal investigation indicated that dispersin is highly immunogenic and can be considered as a vaccine candidate antigen for prevention of EAEC infections. In agreement with our observations, Huang *et al*.[22] suggested that dispersin is immunogenic in a human EAEC challenge model and holds considerable promise as an immunogen. In other part of the study, dispersion and adherent activity of dispersin and anti-disperin *in vitro* were evaluated. We observed that the majority of the EAEC isolates adhered to the cells, but the anti-dispersin antibody could decrease the adherence of EAEC isolates to the cultured cells. The other investigations demonstrated that dispersin acts as an important factor in initial adherence and colonization of EAEC in the intestinal tract[6,9]. The protein also performs an important role in facilitating the movement of bacteria across the surface of the intestinal mucosa, which can subsequently promote the aggregation and adherence. Therefore, our findings indicate that dispersin play a possible role in initial adherence of EAEC isolates to the intestine mucosa, although further studies are needed to demonstrate the precise role of dispersin protein in different stages of EAEC infection.
